# Norepinephrine Is a Major Regulator of Pineal Gland Secretory Activity in the Domestic Goose (*Anser anser*)

**DOI:** 10.3389/fphys.2021.664117

**Published:** 2021-06-02

**Authors:** Natalia Ziółkowska, Bogdan Lewczuk

**Affiliations:** University of Warmia and Mazury in Olsztyn, Olsztyn, Poland

**Keywords:** pineal gland, norepinephrine, melatonin, birds, circadian rhythms

## Abstract

This study determined the effect of norepinephrine and light exposure on melatonin secretion in goose pineal explants. Additionally, it investigated changes in the content of norepinephrine, dopamine, and their metabolites [3,4-dihydroxyphenylacetic acid; vanillylmandelic acid (VMA); homovanillic acid] in goose pineal glands *in vivo* under 12 h of light and 12 h of darkness (LD), a reversed cycle (DL), constant light (LL), and constant darkness (DD). *In vitro* content of melatonin was measured by radioimmunoassay; contents of catecholamines and their metabolites were measured by high-performance liquid chromatography. Exposure of pineal explants to LD or DL established rhythmic melatonin secretion; this rhythm was much better entrained with norepinephrine exposure during photophase than without it. When the explants were kept in LL or DD, the rhythm was abolished, unless NE was administered during natural scotophase of a daily cycle. *In vivo*, norepinephrine and dopamine levels did not display rhythmic changes, but their respective metabolites, HMV and VMA, displayed well-entrained diurnal rhythms. These results indicate that norepinephrine and sympathetic innervation play key roles in regulation of pineal secretory activity in geese, and that pineal levels of VMA and HMV provide precise information about the activity of sympathetic nerve fibers in goose pineal glands.

## Introduction

The pineal gland produces melatonin (MLT), which regulates many physiological processes cued by the environmental light-dark cycle. In birds, MLT regulates locomotor activity, the rhythm of food intake, the function of the immune system, and thermic homeostasis of the body (Gwinner et al., 1987; Chabot and Menaker, 1992, 1994; Rozenboim et al., 1998; Skwarło-Sońta, 1999). Pineal morphology and the mechanisms regulating its secretory activity vary between avian species (Sato, 2001).

There are three principal regulators of MLT synthesis in birds: light received directly by pinealocytes, intrapineal endogenous oscillator, and norepinephrine (NE) released from sympathetic nerve fibers. Most studies on mechanisms regulating MLT secretion have been performed with chickens (Robertson and Takahashi, 1988a,b, Nakahara et al., 1997; Csernus et al., 1998, 1999; Zawilska et al., 2000; Turkowska et al., 2014). In this species, environmental light entrains a diurnal rhythm of MLT secretion in both *in vivo* and *in vitro* conditions; light acts as both a synchronizing factor in MLT production and an inhibitor of its production (Takahashi et al., 1980; Robertson and Takahashi, 1988b; Csernus et al., 2005; Martyniuk et al., 2020). However, rhythmic MLT secretion, albeit of lower amplitude, persists in chicken pinealocytes kept in constant darkness, suggesting that this rhythm is also endogenously driven (Nakahara et al., 1997). In turkeys, direct photoreception enables quick and precise adaptation of the rhythm of MLT secretion to changing light-dark conditions, and an endogenous oscillator that is present in turkey pinealocytes generates a circadian rhythm of MLT secretion in constant light and constant darkness. Thus, in this species, the pineal gland is highly autonomous in the regulation of rhythmic MLT secretion (Prusik and Lewczuk, 2019). In ducks, in contrast to the species mentioned above, when light acts directly on pinealocytes, it does not precisely entrain MLT secretion to the light-dark cycle, and rhythmic MLT production does not persist in constant light or darkness (Prusik et al., 2015).

In the chicken pineal gland, NE is released from sympathetic nerve fibers during the day and inhibits MLT production (Zeman and Herichova, 2011). Changes in NE content in the pineal gland over the diurnal cycle vary between the avian species investigated so far (Cassone et al., 1986; Zawilska et al., 2005; Lewczuk et al., 2014; Adamska et al., 2019). It has been suggested that, in birds, the importance of adrenergic regulation of MLT secretion increases with age (Calvo and Boya, 1979). Moreover, as the studies cited above have shown, regulation of pineal MLT secretion varies between avian species, but to our knowledge, it has not been investigated in geese.

Therefore, our first objective was to establish the effect of light exposure [12 h of light and 12 h of darkness (LD); constant light (LL); constant darkness (DD)] and NE on pineal MLT secretion *in vitro*. Our second objective was to investigate whether goose pinealocytes can adapt their secretory activity to a reversed cycle (DL) *in vitro*. Our third objective was to study the diurnal and circadian changes in the content of catecholamines [NE; dopamine (DA); 3,4-dihydroxyphenylalanine (DOPA)] and their metabolites [3,4-dihydroxyphenylacetic acid (DOPAC); homovanillic acid (HVA); vanillylmandelic acid (VMA)] in the goose pineal gland *in vivo* under LD, DL, LL, and DD.

## Materials and Methods

### Animals and Sampling

All geese were reared from hatching to 12 weeks of age, and during this time, they were kept under a cycle of 12 h photophase and 12 h scotophase (i.e., LD). To provide illumination during photophase (07:00–19:00) at an intensity of 300 lux at floor level, full-spectrum fluorescent lamps were used. During scotophase, animals were kept in darkness, except during the first 2 weeks, when infrared brooding lamps were used throughout the entire light-dark cycle. The birds had free access to standard food and water. To ensure that the animals were not exposed to other light sources during procedures that were performed during scotophase, night vision goggles were used. When the animals were taken for killing, they were hooded, and after euthanasia, the pineal glands were quickly removed and frozen at −75°C until HPLC was performed. The Local Ethical Commission in Olsztyn, Poland approved all procedures (Nr. 11/2009/N).

### *In vitro* Experiment

The 12-weeks-old geese were killed at 08:00, and the pineal glands were immediately removed from the brain, detached from the *dura mater* and choroid plexus, and prepared for superfusion culture.

**FIGURE 1:**
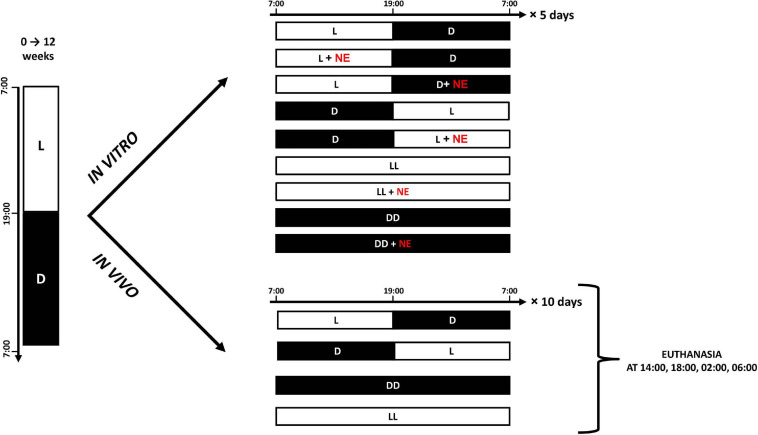
Cartoon presents experimental protocols for *in vitro* and *in vivo* experiments. White bars indicate photophase; black bars indicate scotophase; NE, norepinephrine.

Pineal explants were exposed to different lighting regimes with or without addition of NE, and the concentration of MLT in the superfusion medium was measured with radioimmunoassay (RIA) every 30 min for 6 days. Four explants were cultured in each group, and the conditions of light and NE exposure in each group are shown in Figure 1.

#### Superfusion Culture

The apparatus was the same as in Prusik and Lewczuk (2019). Briefly, single pineal organs were covered with nylon mesh and placed in separate perfusion chambers (0.5 ml volume). The chambers were superfused at a rate of 0.1 ml/min, and the medium was continuously gassed with a mixture of 95% O_2_ and 5% CO_2_. These chambers were covered with translucent plastic sheets during incubation in light (100 lux at the surface of the sheets) and with light-proof sheets during incubation in darkness. Samples of the medium were collected every 30 min for 6 days, and frozen at −20°C until MLT assay. The MLT concentration in the medium was measured by RIA.

#### Chemicals

Powdered Medium 199 containing Earle’s salt and HEPES (Sigma, United States) was used. This powder was dissolved in distilled water, 2.2 g/L of NaHCO_3_ was added, and the pH was adjusted to 7.3 with NaOH before the final preparation was sterilized by filtration. Immediately before use, antibiotic-antimycotic solution (Sigma, United States) was added to the medium; the final concentrations of the antibiotics were as follows: penicillin, 100 IU/ml; streptomycin, 100 μg/ml; and amphotericin B, 0.25 μg/ml).

To create the NE stock solution, bitartrate NE (Sigma-Aldrich) was diluted in ultrapure water (18.2 MΩ, TOC ≤ 5 ppb) to a concentration of 1 mM and sterilized by filtration. After this, the stock solution was diluted in medium.

#### Radioimmunoassay

Anti-melatonin antibody Prospect 6C was kindly provided by Dr. Andrew Foldes, Agriculture Research Western Australia, Australia. RIA was performed as in Prusik and Lewczuk (2019).

### *In vivo* Experiments

Three experiments were performed with a total of 120 geese. First, to investigate diurnal variation in the contents of catecholamines and their precursors and metabolites, 48 geese were used. After the first 12 weeks of life, these geese were killed at 2-h intervals and their pineal glands were removed (Figure 1).

**FIGURE 2:**
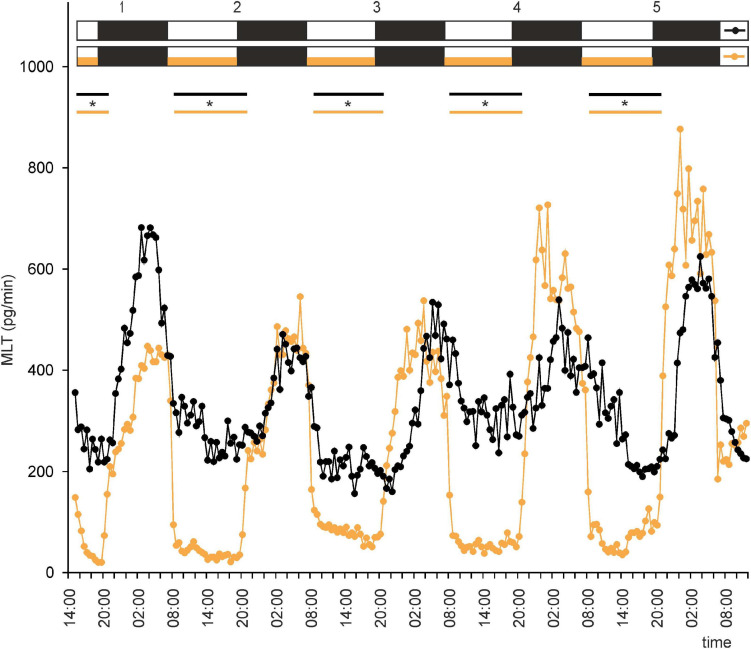
Secretion (means, *n* = 4) of melatonin (MLT) by goose pineal organs incubated under 12 h of light and 12 h of darkness (LD) for 5 days with the presence of NE during photophase (orange line with circles) and without it (black line with circles). White horizontal bars in upper part of image indicate photophase; black bars indicate scotophase; orange bars indicate period of NE treatment. Parallel lines with asterisks show periods, when the means differ significantly (*p* < 0.05) between groups.

Second, in order to determine the effect of DD and LL on the contents of catecholamines, their precursors, and their metabolites, 48 geese were used. After the initial 12 weeks of life, these animals were divided into three groups of 16 and kept under LD, DD, or LL for 10 days, and then killed at 14:00, 18:00, 02:00, and 06:00 (Figure 2). Dim red light with intensity below 3 lux was used during scotophase in LD group and permanently in DD group.

Third, for investigating the effect of a DL cycle on the contents of catecholamines and their precursors and metabolites, 24 geese were used. After the first 12 weeks of life, these were subjected to DL conditions. They were then killed at 14:00 and 02:00 on the first, second, and third days after the change in lighting conditions.

#### Chemicals

To prepare the mobile phase for HPLC, the following chemicals were used: sodium dihydrogen phosphate, disodium EDTA, 1-octanesulfonic acid sodium salt, citric acid, phosphoric acid (J. T. Baker Chemicals, Center Valley, PA, United States), and acetonitrile of gradient-grade HPLC (Merck Millipore, Billerica, MA, United States). NE, DOPA, DA, DOPAC, HVA, VMA, and perchloric acid were obtained from Sigma-Aldrich (St. Louis, MO, United States). Sodium hydroxide was purchased from POCH (Gliwice, Poland), and a Bradford protein assay kit was purchased from Bio-Rad (Hercules, CA, United States). For all procedures, ultrapure water (18.2 MΩ, TOC ≤ 5 ppb) was used, which was freshly prepared using a Milli-Q integral purification system (Merck Millipore, Billerica, MA, United States).

#### Sample Preparation for Catecholamine Assays

First, the frozen pineal glands were weighed, then sonicated (5 × 2 s, 1 W) in 100 μl of ice-cold 0.1 M perchloric acid using a Vibra-Cell VC 70 ultrasonic processor equipped with a 2-mm probe (Sonics and Materials Inc., United States). Next, an ice bath was used to incubate the homogenate for 20 min before centrifugation at 60,000×*g* and 4°C for 15 min (Allegra 64R, Colter Beckman, United States). Finally, the supernatant was transferred to an autosampler vial, and the pellet was frozen at −75°C for catecholamine assays, which were completed within 4 h of sample preparation.

#### Assay of Catecholamines and Their Metabolites

The contents of catecholamines and their metabolites were measured with a chromatographic system as in Lewczuk et al. (2014). Briefly, this system that consisted of a four-channel LPG 3400M pump with a built-in degasser (Dionex, United States), a WPS 3000SL autosampler, and a CoulArray 5600A electrochemical detector equipped with two four-channel coulometric cells 6210 (ESA Inc., United States) was controlled by a Chromeleon 6.8 (Dionex, United States) and CoulArray Data Station 3.10 software (ESA Inc., United States). Twenty microliters of standards or samples were injected onto a 150 × 3.2 mm MG column (ESA, Inc., United States). The column and coulometric cells were kept at 30°C. The mobile phase consisted of a buffer (90 mM sodium phosphate dihydrate, 50 mM citric acid, 1.7 mM 1-octanesulfonic acid sodium salt, and 50 μM disodium EDTA, adjusted to pH 3.05 with phosphoric acid) and acetonitrile, mixed together at a ratio of 94:6 (*v*/*v*). The mobile phase was pumped at a flow rate of 0.5 ml/min. The potentials applied on consecutive electrodes were 150, 200, 350, and 450 mV. Data was acquired and chromatograms were integrated with CoulArray Data Station software, v. 3.10 (ESA Inc., United States). To measure DOPA, DOPAC, DA, and NE contents, the current amperage on a 200-mV electrode was used, and to measure HMV and VMA contents, the current amperage on a 350-mV electrode was used. The duration of analysis was 45 min, which was necessary to prevent 5-HT from interfering during the following assay.

#### Protein Assay

After the pineal homogenates were centrifuged, the pellet was dissolved in 750 μl of 1 M sodium hydroxide. The resulting solution was diluted 1:1 in water then used for measurement of protein content with a Bradford microplate assay kit (BioRad, United States). Solutions of bovine serum albumin in 0.5 M sodium hydroxide were used as standards for preparation of the calibration curve (Lewczuk et al., 2014).

### Statistical Analysis

The effect of time on the level of MLT secretion in each group of explants and the differences in the MLT levels between these groups were analyzed using a one-way analysis of variance followed by a least significant difference (LSD) test. In order to perform these calculations, minutes were expressed as tenths of hours.

To test the differences in the contents of the catecholamines and their metabolites throughout the LD cycle, one-way independent ANOVA was used, followed by Duncan’s *post hoc* test. For testing the differences in the contents of catecholamines and their metabolites between groups (LL, LD, and DD) and between sampling periods within each group (14:00, 18:00, 02:00, and 06:00), two-way independent ANOVA was used, followed by Duncan’s *post hoc* test. To examine the differences in diurnal variation of the contents of catecholamines and their metabolites between the first, second, and third days after reversing the photoperiod (LD–DL), one-way independent ANOVA was used, and then Duncan’s *post hoc* test. Statistica 10.0 (StatSoft, United States) was used for these calculations. Differences were considered significant at *P* < 0.05.

## Results

### *In vitro* Experiments

Under LD, the mean concentration of MLT in the medium was threefold higher during the night than during the day. However, the profiles of MLT secretion were irregular: secretion from individual explants varied substantially from day to day, and there was substantial variation between explants (Supplementary Figures 1, 2A). These results indicate that alternating light and dark phases might not be enough to establish a well-entrained rhythm of MLT secretion by goose pineal glands. Thus, the question arises as to whether the goose pineal gland is photosensitive or whether day/night differences reflect the activity of an endogenous oscillator. The answer to this question is provided by the fact that the pineals incubated in a reversed cycle (DL) adapted their secretory activity to the new lighting conditions over 3 days (Supplementary Figure 2B). As in LD, the daily changes in secretion in DL differed markedly between explants.

**FIGURE 3:**
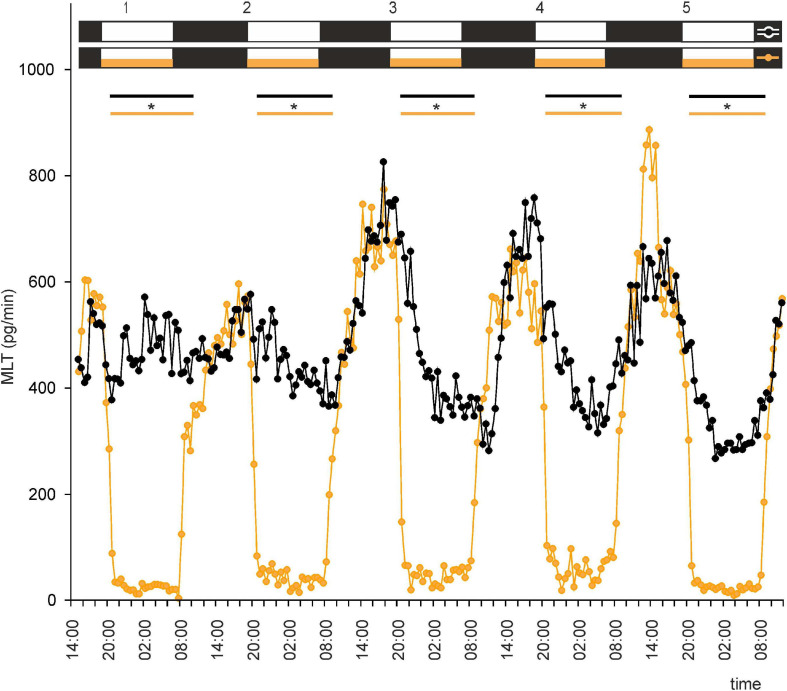
Secretion (means, *n* = 4) of MLT by goose pineal organs incubated under reversed dark-light cycle (DL) for 5 days with the presence of NE during photophase (orange line with circles) and without it (black line with circles). White horizontal bars in upper part of image indicate photophase; black bars indicate scotophase; orange bars indicate period of NE treatment. Parallel lines with asterisks show periods, when the means differ significantly (*p* < 0.05) between groups.

This led us to ask whether NE plays a role in regulation of MLT secretion by goose pinealocytes. We found that, when the pineal explants were in the presence of NE during the photophase of LD or DL cycles, the rhythm of MLT secretion was regular and well entrained in successive cycles, and there was little variation between explants (Figures 2, 3 and Supplementary Figures 2C,D). The amplitude of the cycle (10-fold changes) was more than three times larger than without NE addition (Figures 2, 3). These results indicate that NE plays a major role in the regulation of MLT secretion in the geese pineal organ. In addition, MLT secretion in photophase was lower in the groups incubated with NE addition than in the groups incubated without NE addition (Figures 2, 3). This indicates that NE limits MLT secretion during the time that pineals are exposed to this adrenergic agonist. Moreover, NE addition enabled the goose pineal glands to adapt more quickly to a DL cycle.

**FIGURE 4:**
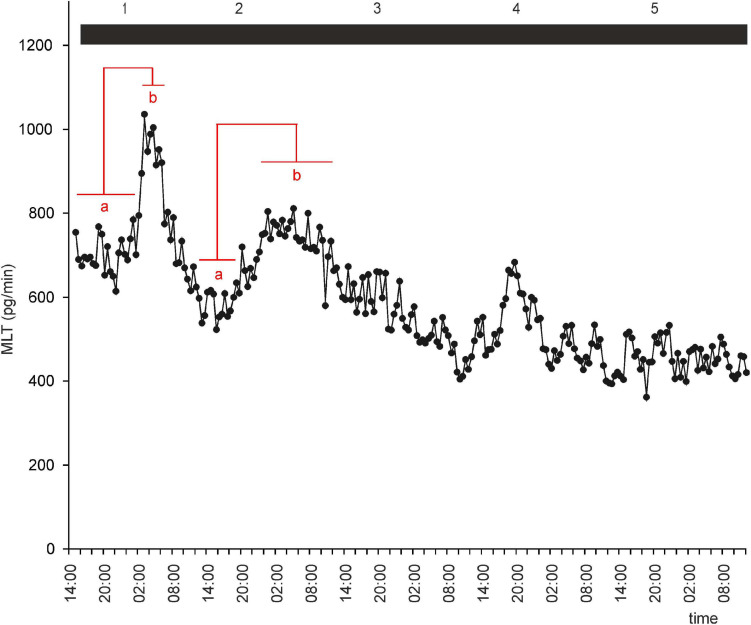
Secretion (means, *n* = 4) of MLT by goose pineal organs incubated in continuous darkness for 5 days. Horizontal red lines show periods, when the means differ significantly (*p* < 0.05) between sampling time-points (repeated measures ANOVA and LSD test).

**FIGURE 5:**
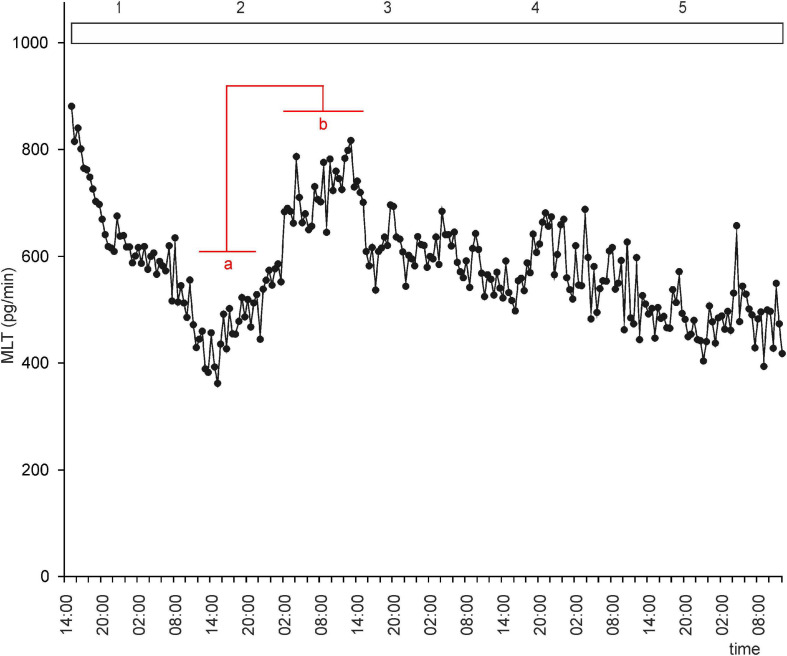
Secretion (means, *n* = 4) of MLT by goose pineal organs incubated in continuous light for 5 days. Horizontal red lines show periods, when the means differ significantly (*p* < 0.05) between sampling time-points (repeated measures ANOVA and LSD test).

To investigate whether there is an endogenous oscillator in goose pineals that can effectively maintain rhythmic secretion of MLT in DD or LL, we incubated pineal explants under these conditions. In both groups of explants, MLT secretion was irregular, with two small peaks during natural night during the first 48 h of the experiment (Figures 4, 5). After 36 h in DD, the MLT level gradually and steadily declined, however, even at the end of experiment was higher than the lowest MLT levels in LD. Together, these results indicate that the endogenous oscillator in goose pinealocytes does not work effectively *in vitro*.

**FIGURE 6:**
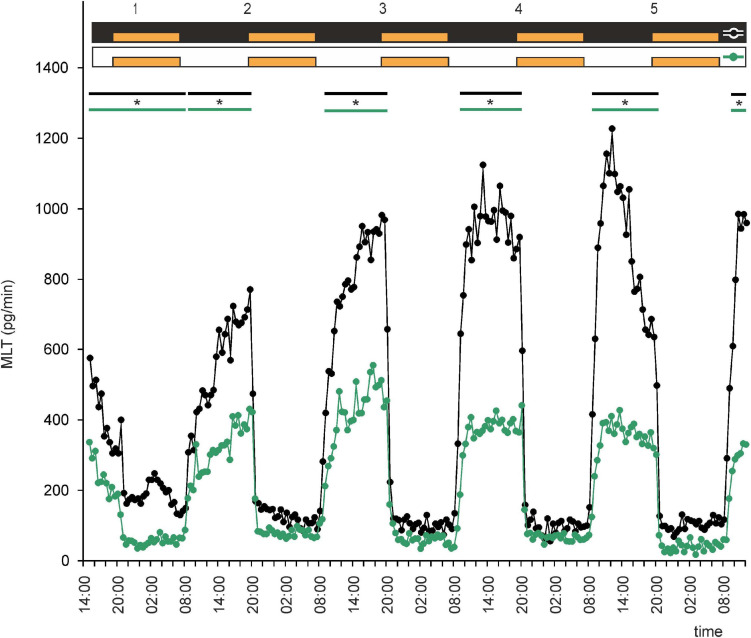
Secretion (means, *n* = 4) of MLT by goose pineal organs incubated under continuous darkness (black line with circles) and continuous light (green line with circles) for 5 days and treated with NE during subjective nights. Orange bars indicate periods of NE treatment. Parallel lines with asterisks show periods, when the means differ significantly (*p* < 0.05) between groups.

We next asked whether administration of NE would establish rhythmic secretion of MLT in LL or DD. Explants incubated under DD with NE administration during subjective nights or under LL with NE administration during the same period secreted MLT in a regular, rhythmic manner (Figure 6 and Supplementary Figures 3A,B). Melatonin levels during subjective day were two times higher in the DD group than in the LL group (Figure 6). Thus, NE administration in LL or DD established rhythmic MLT secretion in these conditions.

**FIGURE 7:**
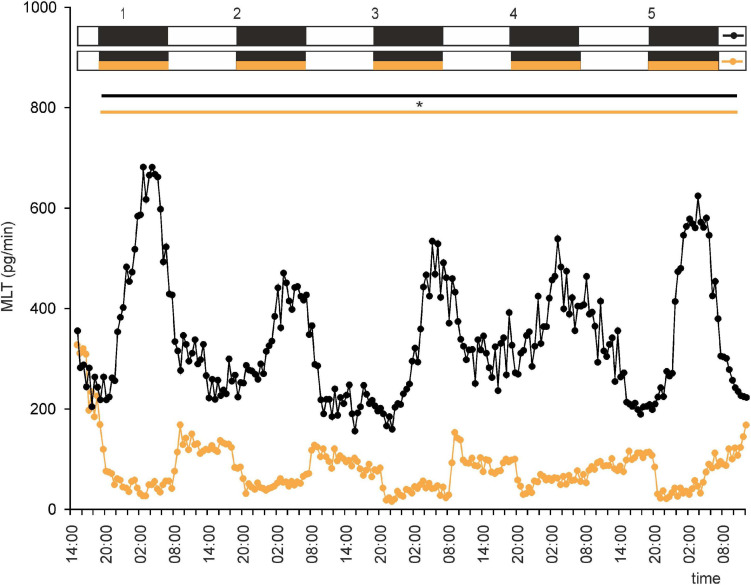
Secretion (means, *n* = 4) of MLT by goose pineal organs incubated under 12 h of light and 12 h of darkness (LD) for 5 days with the presence of NE during scotophase (orange line with circles) and without it (black line with circles). White horizontal bars in upper part of image indicate photophase; black bars indicate scotophase; orange bars indicate period of NE treatment. Parallel lines with asterisk show periods, when the means differ significantly (*p* < 0.05) between groups.

In birds, the nighttime increase in MLT secretion is thought to be due to a combination of stimulation from endogenous oscillators and a lack of light-induced and NE-mediated inhibition. This led us to ask whether NE administration during natural night would abolish rhythmic secretion of MLT. Interestingly, the effect of NE administration was so strong that it reversed the rhythm, with increases in MLT secretion during photophase and decreases during scotophase (Supplementary Figure 3C). However, the amplitude of the rhythm was markedly lower in LD with NE administration during natural night than in LD without NE administration (Figure 7). Moreover, the rate of MLT secretion was much lower in the former than in the latter.

### *In vivo* Experiments

#### Diurnal Variation in Contents of Catecholamines and of Their Precursors and Metabolites

**FIGURE 8:**
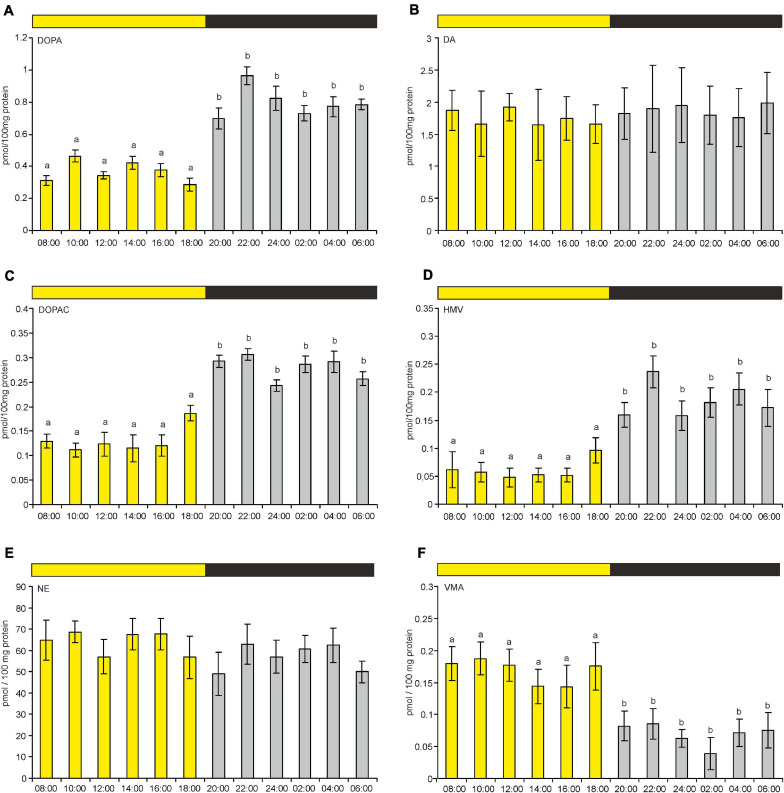
**(A–F)** Content of DOPA, DA, DOPAC, HVA, NE, and VMA in goose pineal organs during a diurnal cycle (means and standard errors, *n* = 4). The values labeled with different letters above the bars differ significantly at *p* ≤ 0.05.

The pineal content of DA and NE did not differ significantly during the LD cycle (Figures 8B,E). However, the contents of their precursors and metabolites did differ significantly between photophase and scotophase. VMA content was significantly higher during photophase than during scotophase (Figure 8F), whereas the contents of DOPA, DOPAC, and HVA were significantly higher during scotophase (Figures 8A,C,D).

#### Effect of a Reversed Light-Dark Cycle on the Contents of Catecholamines and Their Precursors and Metabolites

**FIGURE 9:**
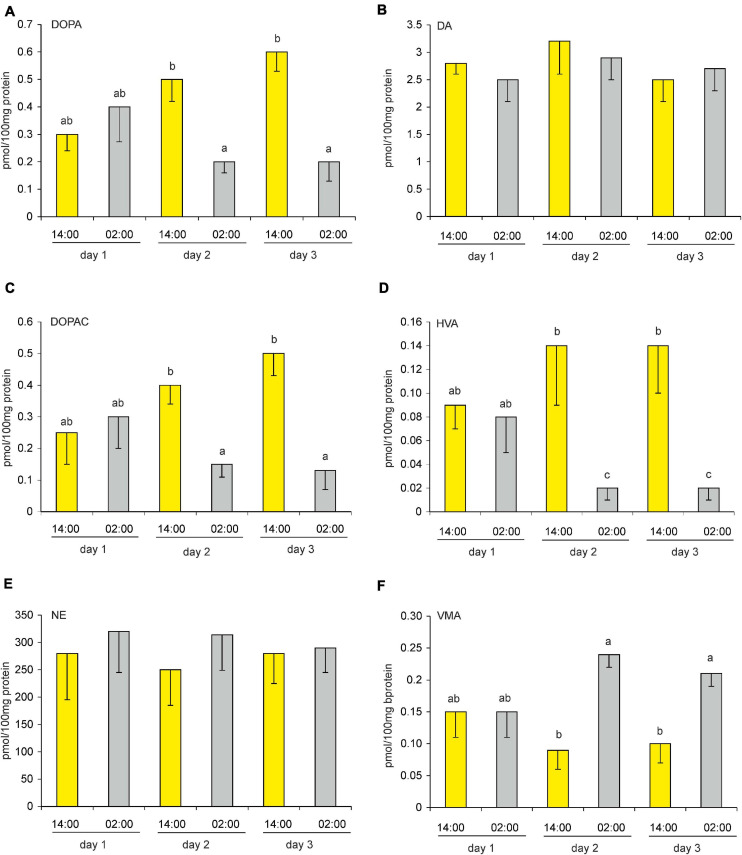
**(A–F)** Effect of a reversed (dark-light) cycle on catecholamines and their metabolites in goose pineal organs. Geese were kept under revered cycle for 1, 2, or 3 days before DOPA, DA, DOPAC, HVA, NE, and VMA contents were measured. Figures show means and standard errors (*n* = 4). Abbreviations and explanations as in Figure 9.

On the first day after reversing the light-dark cycle (i.e., DL), the pineal contents of the catecholamines, their precursors, and their metabolites did not differ significantly between 14:00 and 02:00 (Figures 9A–F). On the second and third days after the reversal, DOPA, DOPAC, HVA, and VMA contents differed significantly between these times, whereas DA and NE contents did not. Similarly to what was observed under an LD cycle, DOPA, DOPAC, and HVA contents were higher during scotophase than during photophase on the second and third days, whereas VMA content was higher during photophase on these days (Figures 9A,C,D,F).

#### Contents of Catecholamines and Their Precursors and Metabolites Under Constant Light and Constant Darkness

**FIGURE 10:**
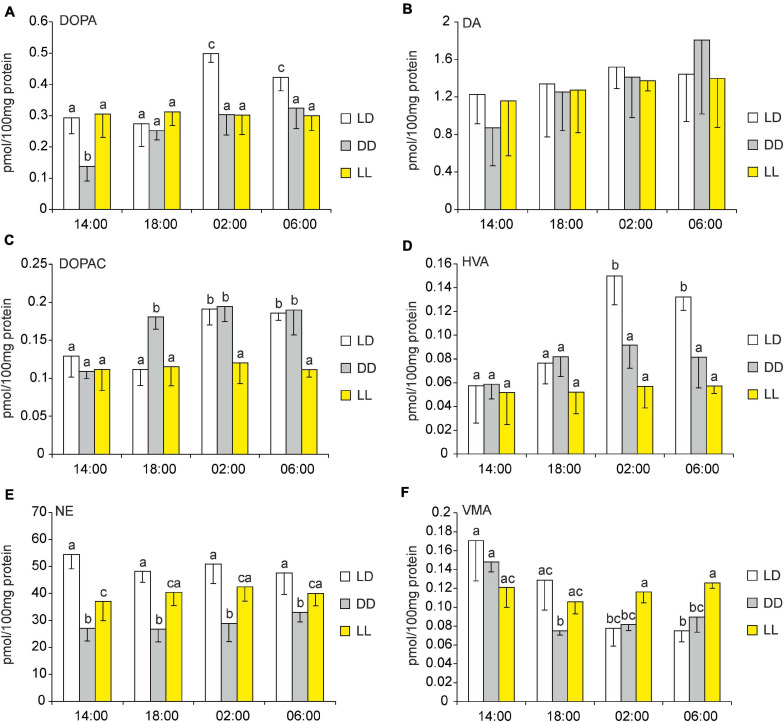
**(A–F)** Effect of constant darkness and constant light on catecholamines and their metabolites in goose pineal organs. **(A–F)** Geese were kept in constant light or constant darkness for 10 days before 3,4-dihydroxyphenylalanine (DOPA), dopamine (DA), 3,4-dihydroxyphenylacetic acid (DOPAC), homovanillic acid (HVA), norepinephrine (NE), and vanillylmandelic acid (VMA) contents were measured. Figures show means and standard errors (*n* = 4). Abbreviations and explanations as in Figure 9.

In the pineals of the control group, i.e., the geese that remained under LD conditions, the changes in the levels of the catecholamines, their precursors, and their metabolites were similar to those reported in Section “Superfusion culture”, above: all substances displayed statistically significant variations over the diurnal cycle, except for DA and NE (Figures 10A–F). Under LL conditions, the levels of these substances showed little variation throughout the circadian cycle. Under DD conditions, the levels of DOPA and DOPAC were significantly lower at 14:00 than at the other sampling times (Figures 10A,C); VMA levels were significantly higher at 14:00 than at the other times (Figure 10F); and the levels of the other substances were similar throughout the cycle. Interestingly, at all sampling times, NE levels were significantly lower in DD than in LD and LL (Figure 10E). Moreover, the level of this catecholamine was significantly lower in LL than in LD at 14:00; at other sampling times, its levels were slightly lower in LL than in LD, but the differences were not statistically significant.

## Discussion

Our results demonstrate that, in most goose pineal organs incubated in superfusion culture under LD conditions, MLT is secreted in diurnal rhythms, with low levels during the day and approximately threefold higher levels at night. However, there were differences between individual explants. In some explants, the rhythm of MLT secretion was irregular and the amplitude was very small. Thus, in geese as in ducks (Prusik et al., 2015), light alone is not sufficient for precise entrainment. In contrast, the rhythm of MLT secretion is well entrained to the phases of the LD cycle in chickens (Takahashi et al., 1980; Robertson and Takahashi, 1988b; Leung, 1991; Csernus et al., 1999), sparrows and pigeons (Murakami et al., 1994), Japanese quail (Underwood et al., 1984), and nestling ducks (Prusik et al., 2015). In turkey pineal glands, this rhythm is also well entrained to the LD cycle, and moreover, the amplitude is much larger than in the glands from other avian species, as MLT levels in turkey pineal organs can be as much as 40-fold higher during the night than during the day (Prusik and Lewczuk, 2019). Comparison of our results and those of Prusik (Prusik et al., 2015) with the results of Murakami et al. (1994); Csernus et al. (1999), and Prusik and Lewczuk (2019) suggests that the role of direct photosensitivity in the regulation of pineal secretory activity is less important in geese and ducks than in turkeys and chickens.

When the goose pineal glands were incubated in DD and LL, the glands secreted MLT in an irregular manner, suggesting that, in this species, endogenous oscillators do not play an important role in rhythmic MLT secretion and that an extra-pineal oscillator is involved in this process. Similarly, Murakami et al. (1994) and Steele et al. (2003) have reported that, when Japanese quail pineal organs were kept in DD *in vitro*, the rhythm of MLT secretion was irregular or abolished, but in animals kept in DD, it persisted, suggesting that an extra-pineal oscillator also plays a role in MLT secretion in this species. However, contrasting results have been obtained with other avian species. In pigeons and sparrows, rhythmic MLT secretion persists for 3–4 days (Murakami et al., 1994), and in turkeys and chickens, it continues for 3–5 days (Robertson and Takahashi, 1988b; Csernus et al., 1998; Prusik and Lewczuk, 2019). It should be noted that these differences between studies may be due to the age of the birds (Prusik et al., 2015; Ziółkowska et al., 2018) and the material used in the perfusion cultures (organs vs dispersed pinealocytes). On the third day of incubation of goose pineal explants under a DL cycle, the explants had adapted their secretory activity to the new lighting conditions, although the amplitude of the rhythm was lower than that of the LD cycle. Similarly, Csernus et al. (1999) and Prusik et al. (2015) have reported that it takes more than 2 days for the pinealocytes of adult ducks and chickens to adapt to reversed lighting conditions. In contrast, the pinealocytes of turkeys and young chickens can adapt to new lighting conditions in 2 days (Csernus et al., 1999; Prusik and Lewczuk, 2019). The effect of reversing the light-dark cycle on the amplitude on MLT secretion differs between avian species. In our geese, the amplitude was lower with a DL cycle than with an LD cycle, as also occurred in young chickens (Csernus et al., 1999). In contrast, in nestling ducks (Prusik et al., 2015) and in turkeys (Prusik and Lewczuk, 2019), the amplitude was similar after cycle reversal, whereas in adult ducks, it was larger after switching to a DL cycle (Prusik et al., 2015).

The effect of NE on MLT production differs substantially between mammals and birds. In mammals, NE is released during the night, and it binds to β- and α-1 adrenoceptors on pinealocytes, activating one of the most important enzymes in MLT synthesis, arylalkylamine-*N*-acetyl-transferase. In the bird species that have been examined so far, NE is released during the day, and it binds to α2-adrenoceptors on pinealocytes and inhibits arylalkylamine-*N*-acetyl-transferase activity and MLT production during the day (Zeman and Herichova, 2011).

Our *in vitro* results indicate that NE has a strong inhibitory effect on MLT secretion in goose pineal organs. Evidence for this conclusion is provided by a number of observations. First, when NE was administered during natural day in LD conditions, the amplitude of the rhythmic variations in MLT secretion was larger than when it was not administered. This increase in amplitude was due to a decrease in the amount of MLT secreted during photophase. Second, NE administration caused immediate adaptation to DL conditions, and the amplitude of MLT secretion in these conditions was also larger than in LD conditions without NE administration. In both LD and DL conditions, MLT secretion during the nadir of the cycle showed less variation when NE was administered than when it was not administered. Finally, NE administration in LL or DD established rhythmic MLT secretion in these conditions, although the amplitude of this rhythm was much lower in LL.

Our *in vitro* results are similar to those observed in a study of the pineal organs of adult and 1-day-old ducks incubated in LD and DL conditions, in which NE administration caused precise adjustment of the rhythm of MLT secretion, a very high amplitude of MLT secretion, and rapid adaptation to the reversed cycle (Prusik et al., 2015). Also, administration of NE to chicken pineal glands increases the amplitude of MLT output *in vivo* (Cassone and Menaker, 1983) and *in vitro* (Zatz and Mullen, 1988; Li and Cassone, 2015). In contrast, in Japanese quail, adrenergic stimulation of the pineal gland may not be as important for MLT secretion because the superior cervical ganglia, where the adrenergic nerve fibers that innervate the pineal gland originate, are not necessary for the pineal MLT rhythm to persist (Barrett and Underwood, 1992). Taken together, our results and those of Prusik et al. (2015) indicate that, in *Anseriformes* birds (ducks and geese), NE and sympathetic innervation play a major role in the regulation of MLT secretion.

Our *in vitro* results led us to investigate whether, in the goose pineal gland *in vivo*, levels of the principal catecholamines, NE and DE, and those of their metabolites, VAM, HMV, and DOPAC, change during the diurnal cycle, and whether prolonged periods of light and darkness can modulate these changes. We found that NE and DA did not display rhythmic changes during the LD cycle in goose pineal glands. This is similar to observations in ducks and 12-weeks-old chickens (Cassone et al., 1986; Lewczuk et al., 2014), but different from observations in younger chickens (Zawilska et al., 2005; Adamska et al., 2019). Lewczuk et al. (2014) reported that, in 14-weeks-old ducks, NE and DA levels did not differ significantly throughout the diurnal cycle. Similarly, Cassone et al. (1986) reported no significant changes in NE content during the diurnal cycle in 12-weeks-old chickens, but they did observe a small peak in DA levels shortly after the onset of darkness. In 2-weeks-old chickens, in contrast, Adamska et al. (2019) found diurnal variation in NE content, with low levels during photophase and high levels during scotophase, but did not observe variation in DA content. With regard to age differences in chickens, Zawilska et al. (2005) reported that the level of NE in the pineal gland increases in the first 2 months of life, and that NE shows rhythmic changes in 4- and 8-weeks-old chickens, but not in younger birds, suggesting that the importance of adrenergic regulation of MLT secretion in this species increases with age. During the process of maturation of avian pineal glands, the photoreceptive structures are thought to be reduced as the sympathetic innervation develops (Calvo and Boya, 1979). Although it is true that sympathetic fibers are present in the quail pineal gland 3 days before hatching (Araki et al., 1992), it has not been determined whether all components of the sympathetic pathway have fully developed at this age or whether NE is released from these fibers. Thus, while the exact details of development of avian pineal-gland innervation still need to be elicited, it does seem likely that the differences between the studies described above may, at least in part, be due to the age of the birds.

Interestingly, while levels of DA and NE did not display rhythmic changes in the goose pineals *in vivo*, the content of the DA precursor (DOPAC), that of the DA metabolite (HMV), and that of the NE metabolite (VMA), did display well-entrained diurnal rhythms. This contrast is likely due to the fact that NE and DE are stored in vesicles in nerve fibers, and only some of the content of these vesicles is released during signal transmission. Therefore, the changes in DOPAC, HVA, and VMA content do not reflect changes in the total pineal content of their precursors, but rather, they likely reflect the different amounts of NE and DA that are released during photophase and scotophase. Indeed, the level of VMA was substantially higher during photophase than scotophase, indicating that NE release is more intense during photophase. Similarly, in 14-weeks-old ducks, the level of VMA is more than twofold higher during photophase (Lewczuk et al., 2014). Our results, together with those from ducks, suggest that levels of VMA and HMV provide precise information about the activity of sympathetic nerve fibers in Anseriformes pineal glands throughout the diurnal cycle. In chickens, however, VMA and HVA content do not vary during diurnal cycles, suggesting that the amount of NE and DE released also does not vary during these cycles (Adamska et al., 2019). These interspecies differences in release of NE and DE would likely be due to differences in sympathetic input from the retina to the pineal gland.

Three of our findings shed light on the regulation of catecholamine synthesis in the goose pineal gland. First, we found that, although the content of NE in this gland does not exhibit a circadian rhythm, it is higher in birds kept in LD and LL than in those kept in DD. This suggests that light deprivation reduces synthesis of this catecholamine. Second, we observed that pineal contents of DOPA, DOPAC, and VMA continue to vary throughout the circadian cycle even after 10 days of light deprivation. Although the pineal gland remains the dominant component of the avian circadian pacemaker (Cassone and Menaker, 1983; Kumar et al., 2004), this result indicates that there may be some kind of internal oscillator that regulates circadian variation in the activity of the adrenergic nerve fibers that innervate the pineal gland. In general, circadian rhythms in birds are controlled by the multiple circadian pacemakers that are entrained by the photoreceptive elements in the central nervous system. The relative importance of these pacemakers varies among the avian species investigated so far, but in each, the core of the circadian system can be identified as the pineal gland, the retinae, and the medial and visual suprachiasmatic nucleus (Trivedi et al., 2017). However, there could also be other oscillators that are responsible for fluctuations in the content of these catecholamines. Third, we found that, in geese kept in LL, there was little or no variation in the pineal content of the catecholamines. This suggests that constant exposure to light inhibits the functioning of the oscillator that regulates the activity of the adrenergic nerve fibers that innervate this gland.

To our knowledge, this is the first study to examine the pineal content of catecholamines and their metabolites in birds subjected to a reversed (i.e., DL) cycle. Our finding that variations in the content of these substances quickly reflect changes in the lighting regime is similar to what we previously observed with regard to variations in the pineal content of indolamines in geese and in ducks (Lewczuk et al., 2014; Ziółkowska et al., 2018). Taken together, these three findings indicate that adrenergic innervation plays an important role in the adaptation of the secretory activity of avian pinealocytes to a changed light-dark cycle.

In summary, our results indicate that NE and adrenergic innervation play key roles in the regulation of pineal secretory activity in geese. They also indicate that, in this species, direct photoreception and endogenous oscillators play less important roles in this regulation, which is similar to what occurs in ducks (Lewczuk et al., 2014; Prusik et al., 2015), but different from what occurs in turkeys and chickens (Takahashi et al., 1980; Robertson and Takahashi, 1988b; Cassone et al., 1990; Csernus et al., 2005; Zawilska et al., 2005; Trivedi et al., 2017). These interspecies dissimilarities may be due to differences in expression of photopigments in the pineal gland, which would be an interesting topic for further investigation. Finally, we found that DA and NE levels do not display rhythmic changes in goose pineals *in vivo*, whereas their respective metabolites, HMV and VMA, do display well-entrained diurnal rhythms. This suggests that VMA and HMV levels provide precise information about the activity of sympathetic nerve fibers in goose pineal glands throughout the diurnal cycle.

## Data Availability Statement

The original contributions presented in the study are included in the article/Supplementary Material, further inquiries can be directed to the corresponding author.

## Ethics Statement

The animal study was reviewed and approved by Local Ethical Commission in Olsztyn, Poland.

## Author Contributions

NZ collected and analyzed the data, performed the experiments, and wrote original draft of the manuscript. BL collected the data, performed the experiments, and reviewed and corrected the draft. Both authors approved the final version of the manuscript.

## Conflict of Interest

The authors declare that the research was conducted in the absence of any commercial or financial relationships that could be construed as a potential conflict of interest.
