# Supplementary material for: Norepinephrine Is a Major Regulator of Pineal Gland Secretory Activity in the Domestic Goose (Anser anser)
Source: Front Physiol. 2021 Jun 2;12:664117. doi: 10.3389/fphys.2021.664117 (PMC8206644; doi:10.3389/fphys.2021.664117)
Supplement: Supplementary file 1 [file Table_1.DOCX]

FIGURE S1. Melatonin (MLT) secretion by individual pineal organs (A and B) incubated under 12 hours of light and 12 hours of darkness (LD) for 5 days. Note differences in a course of MLT secretion between explants.

FIGURE S2. Results of repeated measures ANOVA and LSD test performed on the data on melatonin (MLT) secretion by goose pineal organs. **A.** Explants incubated under 12 hours of light and 12 hours of darkness (LD) for 5 days, **B.** Explants incubated under a reversed dark-light cycle (DL) for 5 days, **C.** Explants incubated under 12 hours of light and 12 hours of darkness (LD) for 5 days and treated with norepinephrine (NE) during photophase, **D.** Explants incubated under a reversed dark-light cycle (DL) for 5 days and treated with norepinephrine (NE) during photophase. Horizontal red lines show periods, when the means differ significantly (p<0.05) between sampling time-points.

FIGURE S3. Results of repeated measures ANOVA and LSD test performed on the data on melatonin (MLT) secretion by goose pineal organs. **A.** Explants incubated under continuous darkness for 5 days and treated with norepinephrine (NE) during subjective nights, **B.** Explants incubated under continuous light for 5 days and treated with NE during subjective nights, **C.** Explants incubated under 12 hours of light and 12 hours of darkness (LD) for 5 days with the presence of NE during scotophase. Horizontal red lines show periods, when the means differ significantly (p<0.05) between sampling time-points.
