# Supplementary figures and images for: Norepinephrine Is a Major Regulator of Pineal Gland Secretory Activity in the Domestic Goose (Anser anser)
Source: Front Physiol. 2021 Jun 2;12:664117. doi: 10.3389/fphys.2021.664117 (PMC8206644; doi:10.3389/fphys.2021.664117)

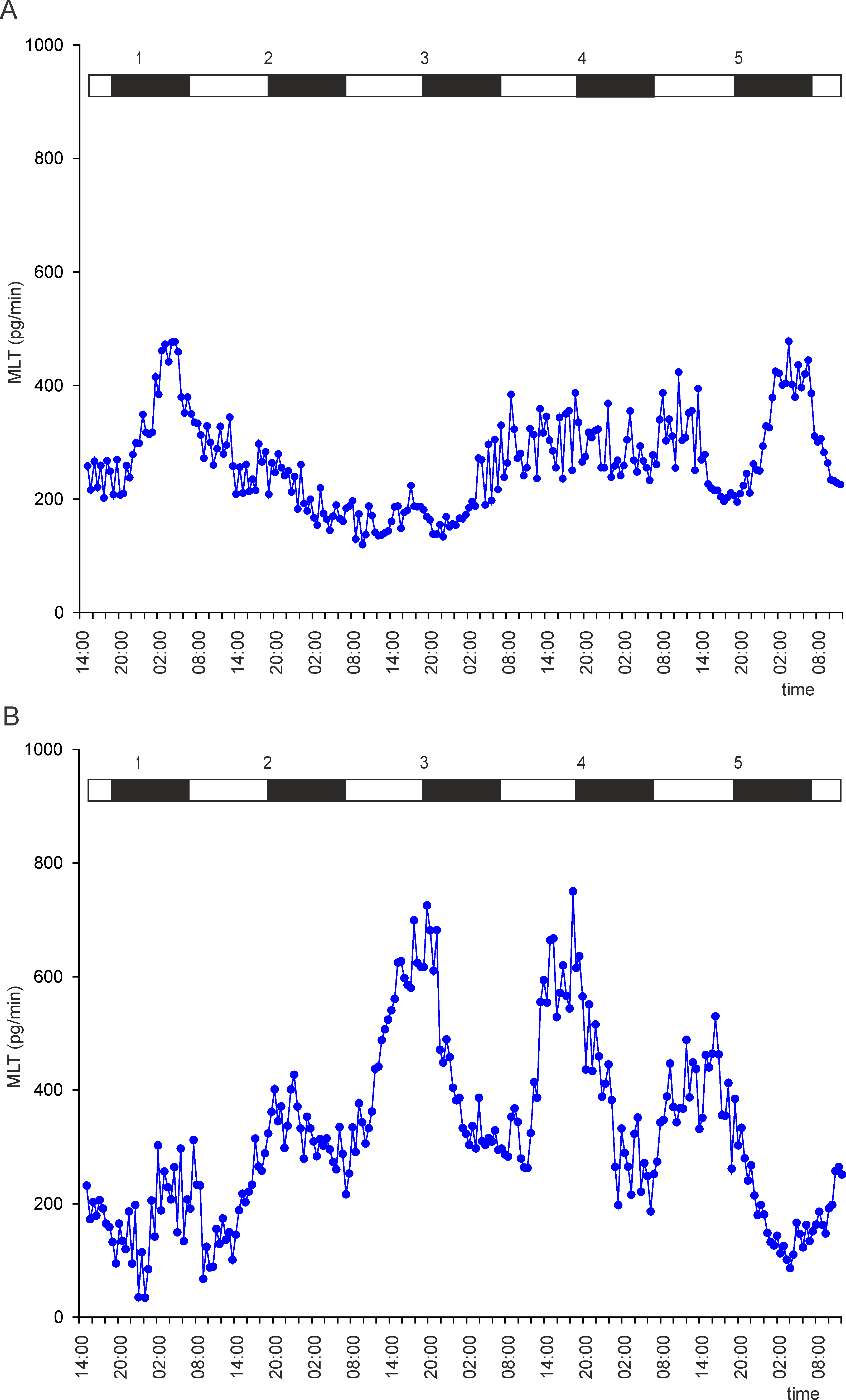

Supplement: Supplementary file 2 [file Image_1.TIF]

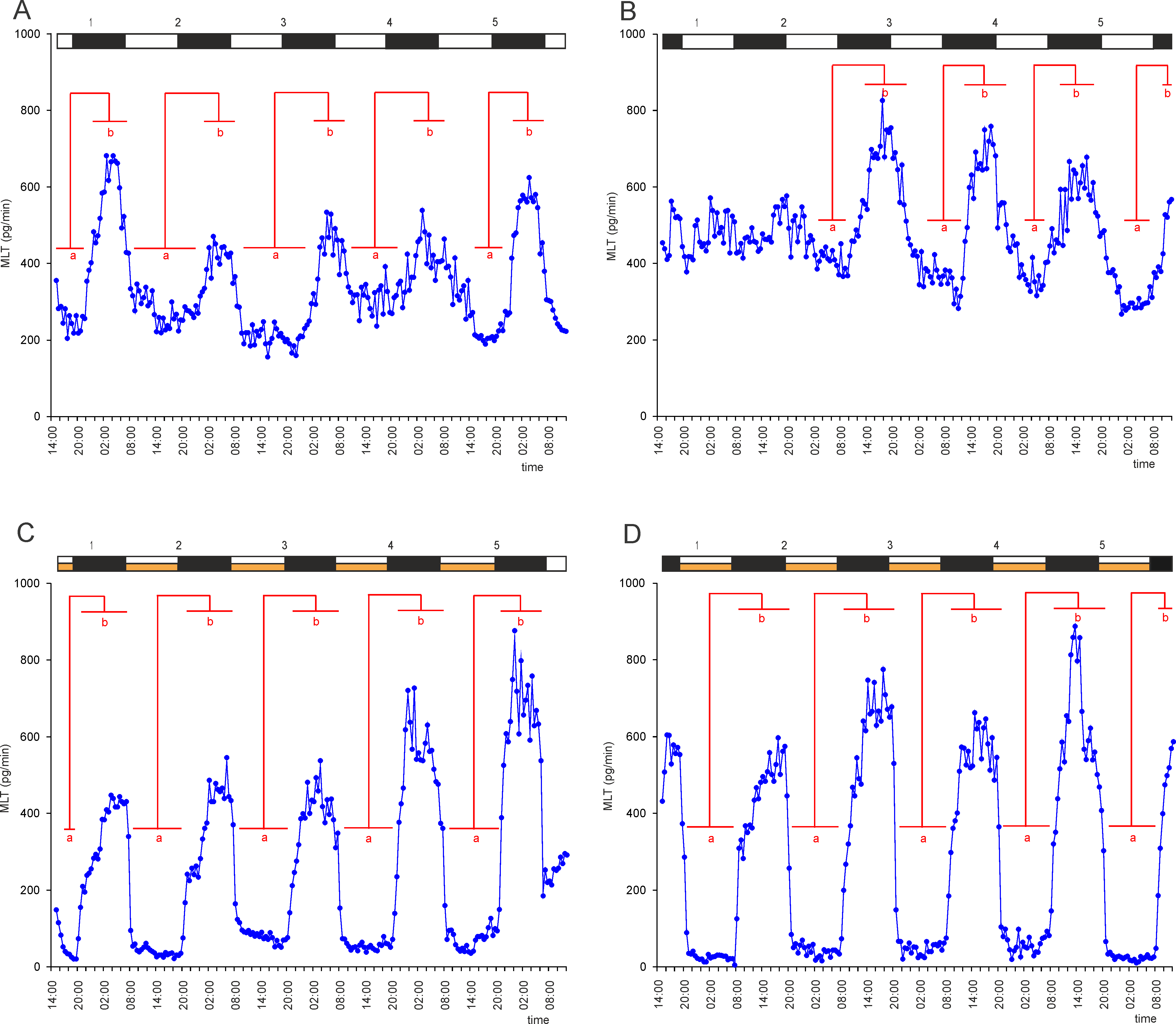

Supplement: Supplementary file 3 [file Image_2.TIF]

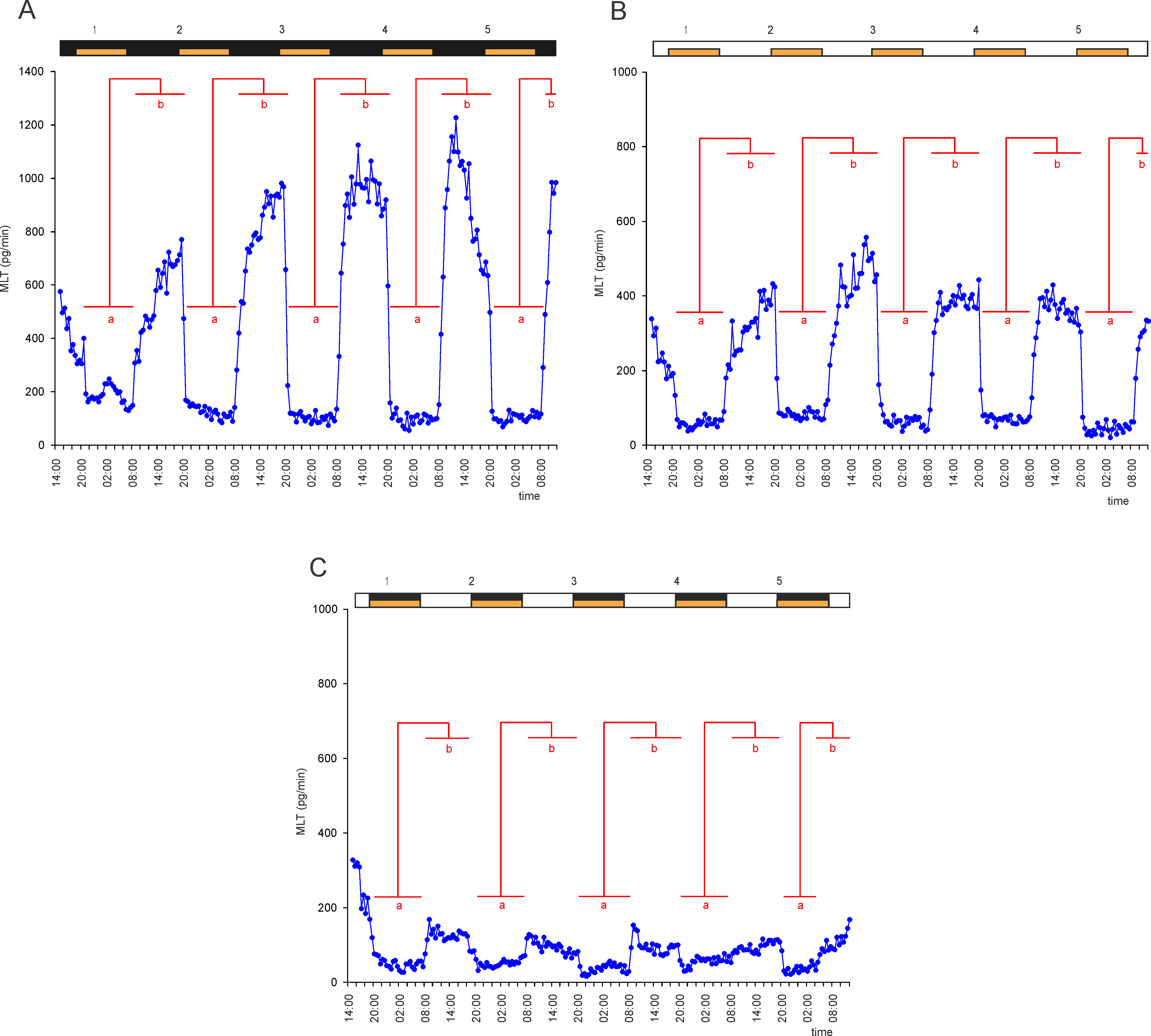

Supplement: Supplementary file 4 [file Image_3.TIF]
